# Constructing “Full-Frequency” Spectra via Moment Constraints for Coupled Cluster Green’s Functions

**DOI:** 10.1021/acs.jctc.2c00670

**Published:** 2022-10-25

**Authors:** Oliver J. Backhouse, George H. Booth

**Affiliations:** Department of Physics, King’s College London, Strand, London WC2R 2LS, U.K.

## Abstract

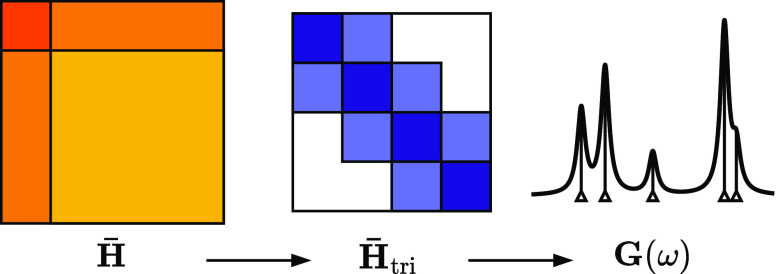

We propose an approach to build “full-frequency” quasiparticle spectra from conservation of a set of static expectation values. These expectation values define the moments of the spectral distribution, resulting in an efficient and systematically improvable expansion. By computing these initial moment constraints at the coupled-cluster level, we demonstrate convergence in both correlated state-specific and full spectral quantities, while requiring a fraction of the effort of traditional Green’s function approaches. Tested across the GW100 benchmark set for charged excitation spectra, we can converge frontier excitations to within the inherent accuracy of the CCSD approximation, while obtaining a simultaneous representation of the entire excitation spectrum at all energy scales.

## 1 Introduction

The single-particle spectrum of a quantum many-body system characterizes the position and weight of all the occupied and unoccupied energy levels present, and its central nature in rationalizing electronic structure means that it is also referred to as the “fundamental” spectrum. This ensures its prominence in numerical methods in electronic structure, and accurately and tractably describing the effect of electron correlation on this quantity is an open and challenging research area.^1−15^ This quantity is directly probed experimentally via photoelectron spectroscopy, as well as other techniques such as scanning tunnelling microscopy.^16−19^ These quantities can be modeled theoretically according to the single particle Green’s function, *G*, expressed as a matrix-valued function of a continuous variable in the real-frequency domain. For an *N*-electron system described by a shifted Hamiltonian *H*_*N*_ = *H* – *E*_0_, the Green’s function can also be expressed in the time domain as
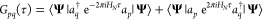
1where the parameter τ = *t* – *t*′, indicating the dependence only on the difference in time for evolution under a time-independent Hamiltonian. The first and second term correspond to the hole (direct photoelectron) and particle (inverse photoelectron) Green’s functions, respectively. The Fourier transform of eq 1 yields the corresponding expression in the frequency domain
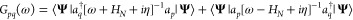
2where η is a small positive broadening factor required to formally regularize this Fourier transform, and defines this (retarded) Green’s function. The fundamental spectrum defining the density of states of the system, as well as the fundamental (quasiparticle) gap, is then characterized as
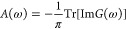
3which tends to a series of delta functions at all ionization potentials and electron affinities of the correlated *N*–body interacting system as η → 0^+^.

At the mean-field level of theory, the ground-state |**Ψ**⟩ is defined by a single (typically Hartree–Fock) reference state determinant |**Φ**⟩, and in the limit of vanishing η, the spectrum of *G*_*pq*_(ω) consists of a series of equally weighted delta functions at the orbital energies of the single-particle Hamiltonian. In this work, we aim to include the effects of correlation on this spectrum, which introduces additional peaks in the spectrum, modifications and rearrangement of spectral intensity between the peaks, and shifting of the position of these energy levels, with important modifications for the described ionization potentials (IPs), electron affinities (EAs), and fundamental gaps of the system.^20^ Specifically, we aim to describe these correlation-driven changes to the spectrum via the coupled-cluster (CC) level of theory.^21−27^ This theory is well-known as the gold standard of quantum chemistry, and has recently seen a revival in its use for describing spectral functions according to the CC Green’s function (CCGF) formalism,^28−35^ which is closely connected to the equation of motion (EOM-CC) formalism^36−38^ and includes recent applications to the description of bandstructures in the solid state and combination with other embedded numerical methods.^32,39−48^

In this work, we describe a numerically simple and low-scaling approach to obtain a systematically improvable approximation to the coupled-cluster single-particle Green’s function across all energy scales, and which can be simply adapted from any equation-of-motion coupled-cluster code. This allows for the full CC Green’s function including off-diagonal elements to be probed for all frequencies, without requiring *a priori* frequency grid definitions on which the function is resolved. Furthermore, this Green’s function and self-energy can be directly obtained as a series of specific energies and spectral weights of all poles in the η → 0^+^ limit, in a fashion similar to recent reformulations of “frequency-free” GW, GF2, and other correlated Greens function methods.^14,15,49−53^ Furthermore, in contrast to some other approaches, this CC Green’s function is not solved for one frequency at a time, nor resolved as a state-specific expansion of successive IPs and EAs.

Instead, a series of moments of the IP and EA spectral distributions are recursively computed at the CC level, which can be formulated as simple expectation values. From these, a Green’s function can be algebraically constructed which ensures that those occupied and unoccupied spectral moments are exactly matched. This also contrasts conceptually with common Lanczos-based approaches which formally conserve a series of moments of the *self-energy*, rather than the Green’s function as done here. We show that this approach can converge a good approximation to the full frequency dynamics of the coupled-cluster Green’s function while saving many orders of magnitude in the number of matrix-vector multiplications of the CC similarity transformed Hamiltonian, compared to traditional approaches based on solving at individual frequencies.^29,31,33−35,45^ Furthermore, this approach is fundamentally adapted for the non-Hermitian nature of the CC Hamiltonian, ensuring that both diagonal and off-diagonal elements of the Green’s function are treated faithfully.

In section 2 we recapitulate the formal perspectives of coupled-cluster Green’s function (CCGF) theory, describing our approach based on a truncated Green’s function moment expansion in section 3. Finally, we numerically compare the convergence of our moment expansion to the traditional “correction vector” approach to CCGF, before analyzing the convergence of CCGF for a common test set of molecular systems for spectral properties (GW100 test set^54^) in section 4.

## 2 Coupled-Cluster Green’s Functions

In this section, we review the formulation of coupled-cluster Green’s function theory, with more details available in ref (35). In coupled cluster, the ground state is parametrized according to an explicitly size-extensive exponential ansatz,^27^ as
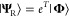
4where the cluster operator *T* can be expanded in terms of particle–hole excitations (where occupied spin–orbitals are denoted *i*, *j*, *k*, ... and virtual spin-orbitals as *a*, *b*, *c*, ...) from a reference state up to a given order
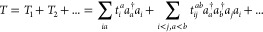
5Since *e*^*T*^ is nonunitary, the bra corresponding to |**Ψ**_R_⟩ = *e*^*T*^|**Φ**⟩ cannot be expressed simply as its adjoint, and instead one must introduce a pair of biorthogonal wave functions required for general expectation values,^36^ with
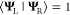
6Typically one expands ⟨**Ψ**_L_| in a linear de-excitation operator Λ
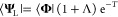
7where the Λ de-excitation operator can be written in a similar truncated fashion to eq 5, as
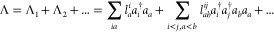
8After insertion of the biorthogonal ground state wave functions (eqs 4 and 7) into the expression for the single particle Green’s function (eq 2), one arrives at the expression for the (retarded) coupled cluster Green’s function
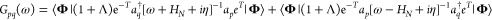
9This can be reformulated in terms of a similarity-transformed normal-ordered Hamiltonian, , where *E*_*CC*_ is the ground-state CC energy. This operator is non-Hermitian, ensuring that the resulting Green’s function is also non-Hermitian, even in the limit η → 0. By noting that *e*^*T*^*e*^–*T*^ = *I* and also introducing the notation for similarity-transformed creation and annihilation operators  and , eq 9 can be rewritten as
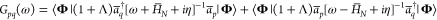
10A pair of explicitly frequency-dependent many-body operators are introduced,
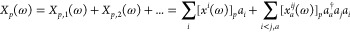
11
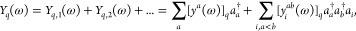
12whose parameters can be optimized at each frequency point to solve the system of linear equations,
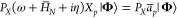
13
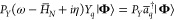
14In these expressions, *P*_*X*_ and *P*_*Y*_ are projection operators onto the appropriate space of electron removal/addition states to ensure well-determined equations, i.e.
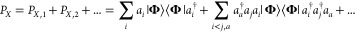
15
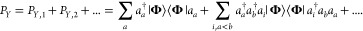
16Once these equations are satisfied for a given frequency, one may evaluate the coupled cluster Green’s function as
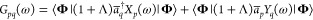
17

The most common coupled cluster singles and doubles (CCSD) method consists of a truncation of *T* to first- and second-order contributions only. Equivalent truncations are then applied to the Λ operator, while the *X*, *Y*, *P*_*X*_, and *P*_*Y*_ operators are truncated to span 1*h*/2*h*1*p* and 1*p*/1*h*2*p* spaces, respectively, as shown in eqs 11 and 12. The choice to truncate the *X* and *Y* operators to these spaces ensures that the fluctuation space describing the singly charged excitation manifold is generally not complete, but instead designed to provide a consistent level of description of both the ground and excited states. However, this truncation results in some expectation values from the Green’s function not matching their analogous properties from ground-state coupled cluster theory. In particular, the Galitskii-Migdal expression of the energy from the Green’s function does not match the ground-state energy of the system at the CCSD level, which requires dynamical fluctuations into the 3*h*2*p* space.^28,29^ This aspect will be considered further in section 4.

By employing iterative linear equation solvers for eqs 13 and 14, the dominant cost in computing the Green’s function is reduced to a series of matrix-vector operations between  and a trial vector of *X*_*p*_(ω) for each frequency value of interest, where explicit expressions for the initial vectors can be found in ref (32), with expressions for the action of  found in ref (30). These matrix-vector products represent the core routines in any IP-EOM-CC and EA-EOM-CC implementations.^30,36,37^ This results in a iterative scaling with system size for CCSD-GF of  for the particle removal (IP) states of eq 13, and  for the particle addition (EA) states of eq 14, where *N*_ω_ is the number of frequency points, and assuming that the full Green’s function is sought. The final step corresponding to eq 17 represents a lower, noniterative  scaling.

To mitigate the overall cost, a number of numerical approximations to full CCGF approaches have been developed, as well as efficient parallel implementations. One such implementation employs a model-order reduction technique to project the linear problem onto a more compact subspace over a chosen set of frequency intervals, resulting in a simplified iterative solution for each frequency.^33^ This method is implemented in the highly parallelized GFCCLib package.^34^ Shee and Zgid propose an alternative method in which the similarity-transformed Hamiltonian is recursively projected into a tridiagonal subspace by means of a biorthogonal Lanczos approach.^32^ From this, the Green’s function can be directly constructed and circumvents the need to explicitly solve the linear equations at each frequency. We believe that this is equivalent to a moment expansion of the effective self-energy of the system.^52,55,56^

Due to difficulties with the biorthogonal nature of the theory, Shee and Zgid determined the off-diagonal elements of the Green’s function via computing contributions from the sum of all pairs of single-particle perturbation operators. Formally this results in an  scaling method if all elements of the full system Greens function matrix are required. However, the authors were primarily applying the approach to compute Green’s functions only over a subspace independent of system size, resulting in a return to  scaling in common with other approaches (and the current work) in this case. The significant advantage of these approaches however is that it removes the dependency of the scaling with the number of frequency points, which substantially reduces the prefactor in the Green’s function construction. We aim to retain this feature in our approach of the next section. Finally, in the context of *ab initio* solids, Kosugi and Matsushita proposed an interpolation scheme for Green’s functions which they apply to CCGF at the level of singles and doubles, to avoid the steep cost with respect to *k*-point sampling. The scheme effectively reduces the computational load by allowing spectra on fine *k*-point meshes to be interpolated from a Fourier transform of spectra on coarser *k*-point meshes.^57^

## 3 Moment-Conserved Coupled-Cluster Green’s Functions

### 3.1 Overview

The approach we present in this work bears similarities to recent works to formulate low-cost approaches to CCGF of Shee et al.^32^ and Peng et al.^33^ described above, and inspired by aspects of the “auxiliary” GF2 approach^14,15,52^ and energy-weighted density matrix embedding theory.^56,58^ In order to remove the dependence of the CCGF with respect to an explicit frequency grid and reduce the cost, we again solve for the coupled-cluster Green’s function at all frequencies in a smaller and systematically improvable subspace compared to the full space in which  is represented. This subspace is constructed independently for the hole (IP) and particle (EA) parts of the Green’s functions, and then combined to a single subspace. A key difference to other approaches is that the coupled-cluster Hamiltonian and CCGF equations are *not* formally projected into an explicitly constructed subspace, as would be common to, e.g., Lanczos methods. Instead, a “fictitious” subspace Hamiltonian is recursively constructed, formulated to guarantee that the particle and hole spectral moments up to a given order of the subspace Green’s function exactly match the ones expected from full CCGF theory. This therefore avoids any direct projection of  in the construction of the subspace Hamiltonian, which only enters via the definition of the initial GF moments of the particle and hole spectral distributions, provided as the constraints in the subspace construction. As the number of moment constraints increases, so does the accuracy of the resulting CCGF approximation, and the size of the constructed subspace.

This perspective of fictitious Hamiltonian subspace construction contrasts with the traditional Krylov subspace approach which (from a Green’s function perspective) is based around conservation of spectral moments of an effective self-energy, rather than the Green’s function moments here. It has been found by the authors in ref (52) (albeit for a different level of theory) that defining a subspace Green’s function based on this conservation of the full system Green’s function moments is a significantly more efficient and rapidly convergent approach than the equivalent effective self-energy truncations, which do not conserve these spectral moments in the resulting Green’s function. The method has a formal  scaling to obtain the full CCSD-GF matrix including off-diagonal elements, and without any dependence on a grid resolution. Furthermore, we show that the approach will also generate an explicit pole representation for the resulting self-energy which characterizes the correlation-driven changes to the underlying mean-field description, within the appropriate non-Hermitian nature of CC theory. Finally, we present results for the approach, and characterize the resulting method in terms of the convergence of the full-frequency Green’s function with respect to number of conserved spectral moments, and the resulting number of EOM matrix-vector multiplications required.

### 3.2 Moment-Conserving Algorithm

We first define the precise definition of these occupied (IP) and virtual (EA) GF spectral moments, which are central to the approach. Within a formal Hermitian theory, these are given for an arbitrary state Ψ as
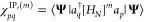
18
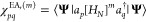
19We denote these as “spectral moments”, as they precisely characterize the moments of the individual IP and EA spectral distributions (up to a sign), as
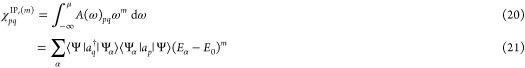
20
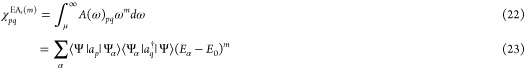
22where μ is the chemical potential, which can be assumed to be the ground state energy of the *N*–electron system (*E*_0_). *A*(ω)_*pq*_ is the matrix valued spectral distribution, which can be related to the (time-ordered) Green’s function as , or given the separation into hole and particle sectors for these equations, can be equivalently considered as the spectra of the lesser and greater Green’s functions for eq 20 and eq 22, respectively. In the sum-over-states representation, α can be considered to sum over all *N* ± 1-electron states with energy *E*_α_. Note that these spectral moments include information on all diagonal and off-diagonal elements. Furthermore, the relationship between the spectral distribution and Green’s function is injective, given the constraints of the Kramers–Kronig relations between the real and imaginary parts of the Green’s function.^20^ Due to the bounded nature of the IPs and EAs and rapid decay of the spectral intensity, the Green’s function can be uniquely determined from the spectral moments as the number of given moments increases. The reconstruction of a spectrum from a set of its moments over the intervals of eqs 20 and 22 in this way is the definition of the “Stieltjes moment problem”, which posed in 1894 the question of reconstructing probability distributions from its moment expansion, and its uniqueness.^59^

These IP and EA spectral moments can also be related to the coefficients of a short-time Taylor expansion of the hole and particle propagation from eq 1, as well as the first 2*m* + 1 terms in the Laurent expansion of the Matsubara Green’s function dynamics, with more details given in ref (58). Finally, linear combinations of these moments up to a given order can characterize the coefficients of various orthogonal polynomial expansions of the separate particle or hole Green’s function in the real-frequency domain, such as Chebyshev expansions which have shown to be rapidly convergent,^60,61^ connecting this approach to any real-frequency polynomial expansion of the Green’s function.

For a mean-field state, all the information on the Green’s function is contained in the *m* = 1 spectral moment (characterizing the Fock matrix), since all higher moments can be found as simple powers of this. In correlated descriptions, these higher moments are not so simply related, and give rise to the additional spectral features as a result of an effective dynamic self-energy. Specializing to coupled-cluster theory, these spectral moments corresponding to the EOM-CC Green’s function can be written in a rigorous diagrammatic fashion (analogous to eq 10), as
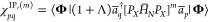
24
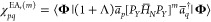
25The presence of the projection operators in this expression is required to match the standard GFCC approach, analogous to the truncation of the *X*(ω) and *Y*(ω) excitation spaces in eqs 11 and 12 to be consistent with the *T* and Λ truncation. These moments can be simply constructed via recursive application of the standard IP- or EA-EOM  matrix-vector product on the initial  and  states,^32^ requiring 2*N*_orb_(*m* – 1) matrix-vector evaluations to construct all moments up to a desired order *m*. Note that the *m* = 0 spectral moment is simply the coupled-cluster one-body density matrix (or one-hole density matrix), and does not require such a matrix-vector product, and that all moments (for both IP and EA) span all occupied and virtual indices.

Once these IP and EA spectral moments up to a given (odd) order of *m* are found, the construction of an effective subspace Hamiltonian whose Green’s function matches these moments can proceed. The initial aim is to formulate separate tridiagonal subspace Hamiltonians for the occupied and virtual spaces, conserving their respective IP and EA spectral moments by construction, with the form
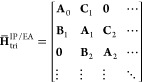
26where each block is an *N*_orb_ × *N*_orb_ matrix. This block tridiagonal form allows for a straightforward connection to the continued fraction representation of the resulting IP and EA Green’s function, via Lanczos-Haydock recursion.^32^ However, the traditional non-Hermitian block Lanczos approach to formulating this matrix is fundamentally changed, such that the recursion is centered around conservation of the resulting spectral moments, rather than just defining a Krylov subspace of higher powers of .^62^ This is adapted from the Hermitian formulation of ref (56) and fundamental work on the Stieltjes moment problem to work with the non-Hermitian moments of CC theory.^63,64^

First, the moments are orthogonalized under the metric of the zeroth moment (transforming to the natural orbital basis), independently for the IP and EA sectors (where explicit indices are dropped in subsequent equations for clarity, with all quantities considered as *N*_orb_ × *N*_orb_ matrices for both the IP and EA sectors), as
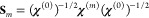
27Following this, the elements of eq 26 are constructed according to the recursive formulas
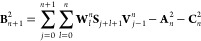
28
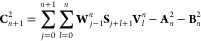
29
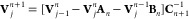
30
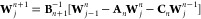
31
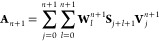
32where the coefficients **V** and **W** satisfy
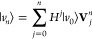
33
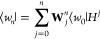
34It must therefore follow that the initial coefficients are identity,
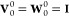
35Traversing the recursive formulas in eqs 28–35 results in a maximum number of  blocks. The coarse-grained excitation energies of each IP and EA sector can therefore be obtained via eigenvalue decomposition of eq 26
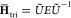
36where the first *N*_orb_ rows of eigenvectors  can be back-transformed from the orthogonal basis to give left- and right-hand transition amplitudes, corresponding to the weight of each excitation over the physical degrees of freedom, as
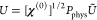
37
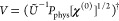
38The projection into the physical space is defined as
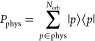
39where *p* enumerate physical states, represented by molecular orbitals at the Hartree–Fock level. The energies, *E*, and vectors, {*U*, *V*}, of these states are then sufficient to recover the original moments of the Green’s function, as
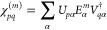
40where α now runs over excitations resulting from both the hole and particle Hamiltonian construction, and *p*, *q* run over the “physical” space of MOs. Critically, these spectral moments will exactly reproduce the original CC spectral moments of eqs 24 and 25 provided, by construction to numerical precision. However, we can now also find a full spectrum on an arbitrary grid of frequency points, for which these moments provide the constraints, as
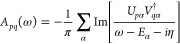
41The number of poles in the approximate spectrum (indexed by α in eq 41, spanning both occupied and virtual states) will grow as the number of provided moments increases, as (at most) *N*_orb_(*m* + 1), compared to the number of mean-field excitations of simply *N*_orb_. These additional states describe the correlation-induced splitting and adjustment of spectral intensity, emergence of quasiparticle lifetimes, and introduction of satellite peaks in the spectrum. Since each recursion of the modified block Lanczos iterations requires two additional GF moments as input, we define the approach via an integer *n*, which conserved all moments 0 ≤ *m* ≤ 2*n* + 1, giving the hierarchy of approximations we denote GF(*n*). While this final “moment-constrained” Green’s function can not be simply written in terms of a connected diagrammatic expansion, the moments of its spectral distribution will be rigorously diagrammatic by construction, which will therefore tend to the exact diagrammatic Green’s function of coupled-cluster theory as *n* is increased.

This algorithm therefore provides a systematic series of approximations, with inclusion of each additional order of *n* requiring further computational effort, but yielding a Green’s function which is closer to the exact dynamic limit. An increase of *n* by one provides an additional (up to) 2*N*_orb_ poles in the quasiparticle spectrum and requires an additional two moments in each sector. The algorithm presented scales only cubically with system size (once the spectral moments have been computed, which is itself  per moment at the level of CCSD), but is capable of producing a spectrum of arbitrary resolution in frequency space. This is a significant improvement upon conventional algorithms which may require multiple evaluations of an  step at each frequency point of interest, constraining the frequency-resolution by available computational resources. However, if very accurate resolution of high-frequency dynamics at the level of CCSD is required, then the moment expansion may not be the most efficient approach compared to a direct frequency-domain targeting algorithm,^28,29,33^ as very high moments may start to be beset by numerical difficulties, which likely require further developments (e.g., reorthogonalization steps) to resolve.

### 3.3 Explicit Self-Energy Construction

One may also wish to algebraically cast the excitations of this spectrum back into a set of static “auxiliary” degrees of freedom which, when folded down into frequency space over “physical” degrees of freedom (e.g., the MO space), represent the effect of a dynamic self-energy.^15,49,50,53,65,66^ This gives a pole representation of the self-energy required to achieve the spectrum of eq 41, without resorting to an explicit Dyson equation requiring a potentially ill-conditioned and costly inversion of the Green’s function at each frequency. To do this, we need to combine the particle and hole excitations into a single Hamiltonian, such that its diagonalization gives the eigenenergies given by *E*_α_, and where the projections of the corresponding eigenvectors onto the physical space give back *U* and *V*. The self-energy can then be represented as the part of this Hamiltonian external to the physical space (describing the augmentation of the MO space due to the self-energy).

First we define vectors spanning the physical space consisting of the projections of the excitations on the MO space, concatenating the vectors for both the IPs and EAs of eqs 37 and 38 as
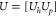
42
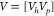
43which have dimension *N*_orb_ × (*L* + *M*), where *L* and *M* are the sizes of the compressed IP and EA spaces, respectively. One can then construct additional *L* + *M* – *N*_orb_ rows of these matrices, in order to define augmented (square), full-rank *U*_*c*_ and *V*_*c*_ matrices of dimension (*L* + *M*) × (*L* + *M*), while maintaining their projection onto the initial *N*_orb_ × *N*_orb_ physical subspace as given by eqs 42 and 43. This can be achieved using any complete biorthogonal basis which does not change the existing vectors in the physical space, e.g., via a two-sided Gram-Schmidt, or using the eigenvectors corresponding to the non-null-space of **I** – *U*^†^*V*. These vectors and the IP and EA excitation energies can then form a biorthogonal eigenbasis for a new Hamiltonian with the desired properties, spanning the “physical” MO space coupled to an external space, which has exactly the spectrum of eq 41 when projected back into the physical space. We can define this Hamiltonian as
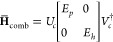
44In order to obtain an explicit pole representation of this effective self-energy,  must be rotated into a representation in which the nonphysical (“auxiliary”) subspace is diagonal, using the eigenvectors of , where *P*_ext_ = 1 – *P*_phys_, and resulting in the (*L* + *M* × *L* + *M*) matrix, . The energies of the poles of the resulting effective self-energy are denoted ϵ, obtained from the diagonal of , with the left- and right-hand residues as the vectors  and , respectively. This allows the explicit self-energy to be written as
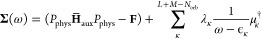
45where *F* is the original Fock matrix in the physical space, and κ runs over the states in the external space. Similarly, the Green’s function can then be written via a Dyson equation as
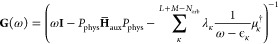
46where the spectrum of eq 46 above will match that of eq 41, with the distinction being that it arises from the diagonalization of a single Hamiltonian, allowing for an explicit self-energy to extracted, rather than from a combination of separate particle and hole Hamiltonians.

One advantage of this explicit self-energy construction is that Fermi liquid parameters such as effective masses and renormalization factors of individual states can be found, which characterize the magnitude of the correlation-driven changes to low-energy excitations, despite not being formal observables themselves. While in condensed matter they can be used to define interaction-driven quantum phase transitions, in molecular and finite systems they can act as a useful proxy for defining the effect of correlations on a specific (molecular) energy level. This quasiparticle renormalization factor for a given MO, *i*, can be written as
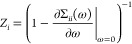
47When the explicit form of eq 45 is considered, this reduces to
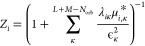
48This renormalization factor, *Z*_*i*_, takes values between zero and one, where one indicates that the correlations have not changed the state at all from its original mean-field description (Hartree–Fock), while smaller values indicate that the stronger correlations have increasingly shifted the energy of the state. We note that for finite systems, this renormalization factor loses its precise motivation in terms of the changing character of electronic structure at the Fermi surface of a metal. However, as a heuristic for the strength of correlation-driven changes to the frontier excitations about the chemical potential, it is still a valid diagnostic. To characterize correlation-driven changes to higher-energy states away from the chemical potential, the derivative of the self-energy at different energies (i.e., the Fock MO energy of the orbital in question) can be used instead.

### 3.4 Technical Details

We have implemented the present algorithm in the PySCF package,^67,68^ where we look to incorporate it as a public-accessible functionality in the near future. The code supports MPI parallelism, distributing the work required to compute the matrix-vector product between MPI processes. Within each MPI process, the matrix-vector product builds on the existing PySCF EOM-CC functionality,^67−69^ which already supports OpenMP parallelism, and we therefore achieve a hybrid parallel algorithm, where each MPI rank is a separate physical node, and OpenMP communication within the nodes, resulting in an effective implementation in high-performance computing settings while retaining a lightweight code base. Our order of operations is designed to reduce communication of large arrays, whereby a job with *K* available MPI processes (generally nodes) proceeds as

1. Compute hole right-hand side  and particle right-hand side  on all MPI processes.
2. Compute hole left-hand side  and particle left-hand side  up to desired moment order, where the effort is distributed among the MO indices of *p*, assigned to process (*p* mod  *K*).
3. Contract left- and right-hand sides for hole and particle cases according to eqs 24 and 25 for elements  on process (*p* mod  *K*).
4. Perform a reduction operation to gather χ between all processes.

This algorithm is therefore embarrassingly parallel for the computation of the moments, assuming the ability to perfectly distribute the load over the MO indices on each MPI rank. With this algorithm we have been able to perform calculations on the full GW100 benchmark set in a def2-TZVPP basis set (see section 4.4), with the largest system in this set having 411 orbitals and 78 correlated electrons. The total run time for the calculation of GF(0) through GF(5) for this system was approximately 2 h on 32 nodes, where each node (consisting of two AMD 7742, 2.25 GHz, 64-core CPUs) was assigned a separate MPI process.

## 4 Results and Discussion

### 4.1 Proof of Principle

**Figure 1:**
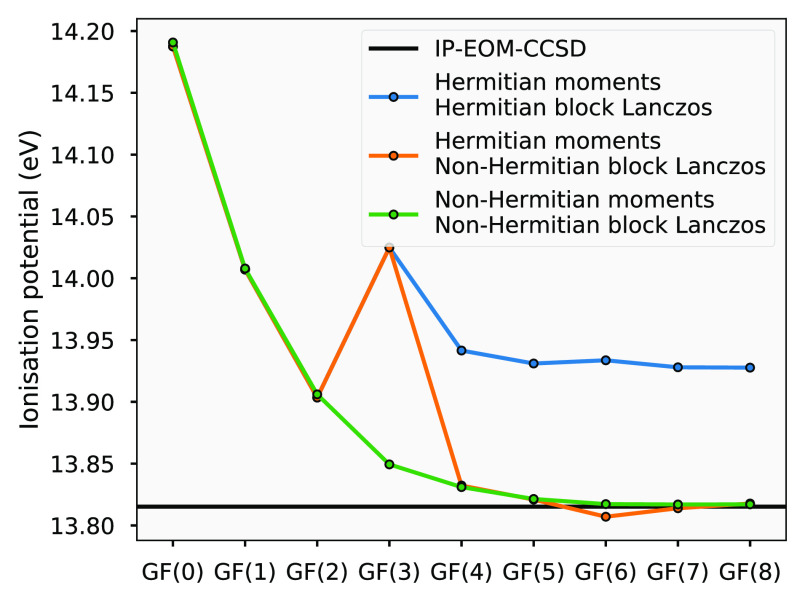
Comparison of the convergence in the ionization potential for a CO molecule (bond length of 1.1314 ) in a cc-pVDZ basis between the Hermitian and non-Hermitian block Lanczos recursion, showing the correct convergence of the non-Hermitian form to the exact IP-EOM-CCSD ionization potential, and incorrect convergence or numerical errors for Hermitized versions. Note that the HOMO energy at the level of the Hartree–Fock starting point is 19.12 eV, showing that even at the lowest levels of moment conservation, significant correlation-driven changes to the IP are found.

In Figure 1, we show convergence of the (first) ionization potential of carbon monoxide in a cc-pVDZ basis, with increasing orders of CCSD Green’s function spectral moment conservation. We stress again that the approach does not directly target this IP, which is more common to “state-specific” approaches to computing excited states in, e.g., ADC or EOM methods,^5,36^ but instead the method involves satisfaction of moment constraints which are integrated quantities over all excitations in the spectrum (eqs 20–23), and therefore convergence of any single excitation is expected to be a stern test. Nevertheless, the aim is to systematically converge to all excitations in the IP/EA-EOM-CCSD spectrum.

We also compare three different variants of the algorithm; the one outlined in section 3, one where we modify each moment to the nearest Hermitian form (in a least-squares sense) before computation of the resulting spectrum (as ), and one where the moments are Hermitized *and* the algorithm of section 3 is constrained to be Hermitian (i.e.,  in eq 26). Note that the emergence of non-Hermitian moments is a feature of the nonunitary nature of CC theory used in the moment construction, with other levels of theory (and the “exact” moments) expected to be Hermitian by construction. The use of a Hermitian (real-valued) block Lanczos recursion of eqs 28–32 requires **B**^2^ to be positively semidefined in order to compute **B**, and positively defined to compute **B**^–1^. Any space corresponding to negative (or zero) eigenvalues in **B**^2^ must therefore be discarded, resulting in the possibility of loss of flexibility in the resulting effective Hamiltonian. In the non-Hermitian recursion, **B** and indeed **C** are allowed to be complex and therefore no such states need be discarded, except any null space when inverting the matrices.

The error due to the approximation of Hermiticity in the EOM-CCSD moments themselves, while still allowing **B**, **B**^–1^, **C**, and **C**^–1^ to be complex, does not produce errors as large in magnitude as the real-valued constraint. This is in agreement with the claim of Shee and Zgid that the error between the exact and symmetric part of the coupled cluster Green’s function is generally small.^32^ Despite this, we can observe that the convergence of the excitations in the case of the complex-valued recursion and Hermitised moments is less rigorous, and there is more sensitive to numerical error. Furthermore, manual Hermitisation of the moments does not necessarily result in a matrix that is guaranteed to have the correct definite structure, causal Green’s function, and self-energy (again, a feature of CC theory). We note that Figure 1 is an example selected where the ill behavior of the Hermitised moments is particularly large, and not all systems have such a large discrepancy between the Hermitian and non-Hermitian variants. Nevertheless, we can clearly demonstrate systematic convergence to the IP-EOM-CCSD ionization potential with the appropriate non-Hermitian recursions.

### 4.2 Full Spectrum Convergence

**Figure 2:**
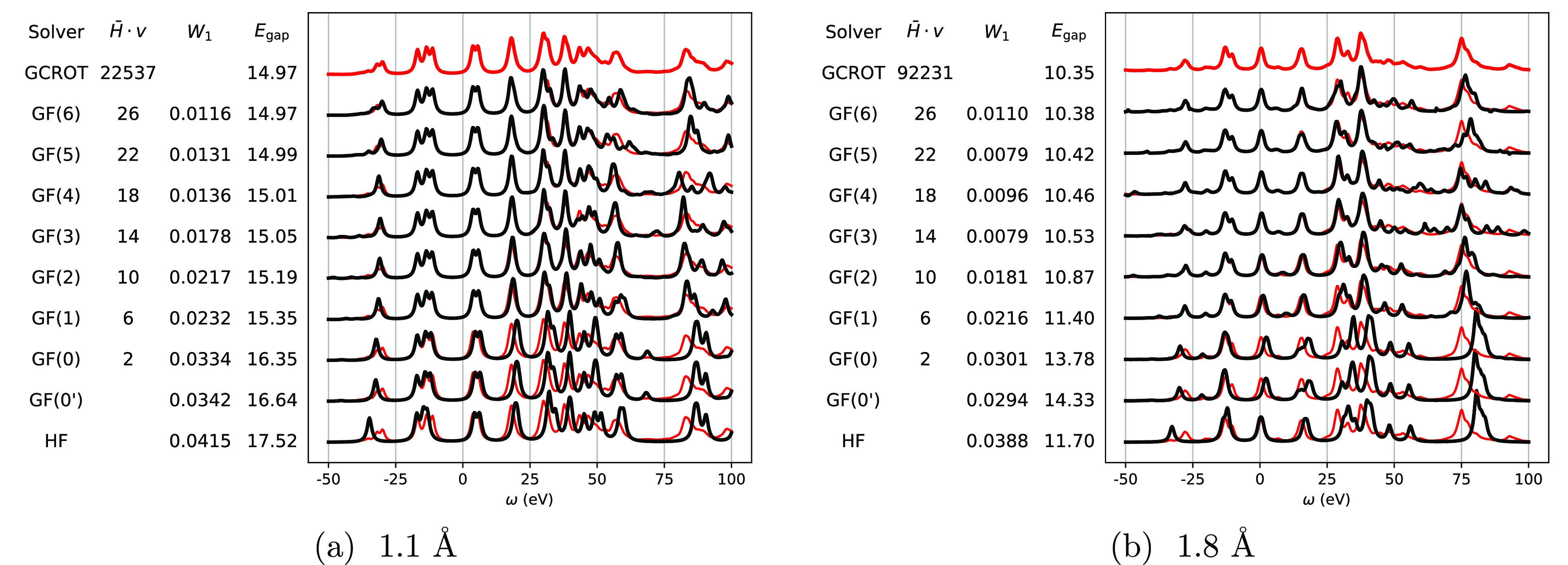
Comparison of theoretical photoelectron spectra computed using the recursive GF moment conservation (presented in section 3 in black), and a more conventional GF-CCSD approach using the GCROT algorithm (in red under-laying all spectra), for water with a bond length of (a) 1.1 Å and (b) 1.8 Å in a cc-pVDZ basis. The notation GF(*n*) indicates the number of iterations of the recursion algorithm performed, conserving all GF moments up to order 0 ≤ *m* ≤ 2*n* + 1. GF(0′) corresponds to a modified GF(0) approximation where the moments are exactly computed via the reduced density matrices (section 4.3). Also shown is the spectrum at the mean-field (Hartree–Fock) level. The labels also give the number of matrix-vector products per orbitals required to produce the spectrum, where the GCROT result depends on the number of frequency points *N*_ω_, which was selected to be 512 in this example with a broadening parameter η of 1.0 eV (the other GF results are artificially broadened with the same broadening). The Wasserstein metric *W*_1_ between each of the spectra and the GCROT spectrum is shown, indicating the their fit to the true GF-CCSD spectrum. The value of the gap for each method is also included, with the chemical potential at the zero frequency.

The strength of the method (over, e.g., an EOM approach) will likely be found in the fact that the entire excitation spectrum can be converged (on the real-frequency axis) to a good accuracy simultaneously, with arbitrarily small broadening. To demonstrate this, in Figure 2 we show the convergence of the full spectrum with increasing numbers of moments, to a more conventional “correction-vector” implementation of CCGF as outlined in section 2 using the GCROT algorithm to solve the linear equations of eqs 13 and 14 at each frequency point of interest and with a fixed broadening (η) of the line shapes.^70−73^ A water molecule in a cc-pVDZ basis was used with a total of 24 orbitals, in both an equilibrium and stretched (more strongly correlated) configuration. The number associated with the labels gives the required number of matrix-vector computations per orbital between  and an arbitrary state vector, which dominates the computational cost. This number scales with the number of frequency points *N*_ω_ only in the case of the conventional (GCROT) CCGF calculation, with *N*_ω_ = 512 used in this plot and broadening to regularize the linear equations of 1.0 eV. The number of required matrix-vector products for a conventional calculation is typically considerably larger than our modified block Lanczos recursion approach to building a representative Hamiltonian from the GF moments, with all “moment-conserving” spectra shown computed for less than the average cost of a *single* frequency point in the traditional frequency-domain approach.

The low-energy excitations around the chemical potential are found to rapidly converge as the numbers of moments conserved increases, with the quasiparticle gap (*E*_gap_) shown to converge to less than 0.1 eV error compared to the “exact” EOM-CCSD one by GF(4) in both correlation regimes (corroborated more broadly by the test set of section 4.4, and which is roughly the intrinsic accuracy of the CCSD method). The only anomalous point is the GF(0) result in the more strongly correlated regime, where the lack of spectral information in the first two hole and particle moments results in a gap which is in error by even more than the initial Hartree–Fock estimate. The importance of correlations in modifying the Hartree–Fock energy levels in the stretched case compared to the equilibrium molecule can be quantified by considering the quasiparticle renormalization factor for the HOMO state (see eq 48). For the equilibrium case, *Z*_HOMO_ = 0.93, while in the stretched case it is *Z*_HOMO_ = 0.27, indicating a significant a qualitative change in the HOMO state upon stretching of the bonds. Individual higher-energy excitations are found to converge more slowly, despite the broad trends of spectral density over higher energies being well reproduced also by GF(4). To quantify this accuracy over the full spectral range, we also provide the Wasserstein (or “earthmover”) metric (*W*_1_) between each spectrum and the exact CCGF one, which appropriately characterizes the shift in overall spectral weight between two probability distributions. This is found to converge in an almost entirely monotonic fashion with the number of conserved moments, demonstrating the systematic convergence over the whole spectral range.

A considerable advantage of the GF moment approach is that an explicit pole structure of both the overall approximation to the GF and self-energy is produced, allowing one to adjust the plotting parameters such as the number of frequency points *N*_ω_, and the broadening parameters η. A traditional CCGF correction vector algorithm requires one to select these parameters beforehand and their adjustment requires new calculations. We note that correction vector algorithms are also more difficult to converge around the excitation energies, requiring substantially more iterations as the condition number becomes larger and the linear equations of eqs 13 and 14 become increasingly singular. While these are not problems in the present algorithm, numerical difficulties exist in a different limit. In exact-precision arithmetic the limit of large numbers of moments should exactly reproduce the exact CCGF result (formally the complete limit for CCSD will scale as  moments). However, floating-point arithmetic makes very large moments difficult to work with, as successively higher powers of  are required. Additionally, a loss in biorthogonality between the left- and right-hand Lanczos block vectors is observed at high moment numbers, which may introduce further errors within finite-precision arithmetic. We consider the improvement of the numerical accuracy and maintenance of biorthogonality to be out of the scope of this current work, where we do not extend to very high moment numbers, and future work will look to remedy this.

**Figure 3:**
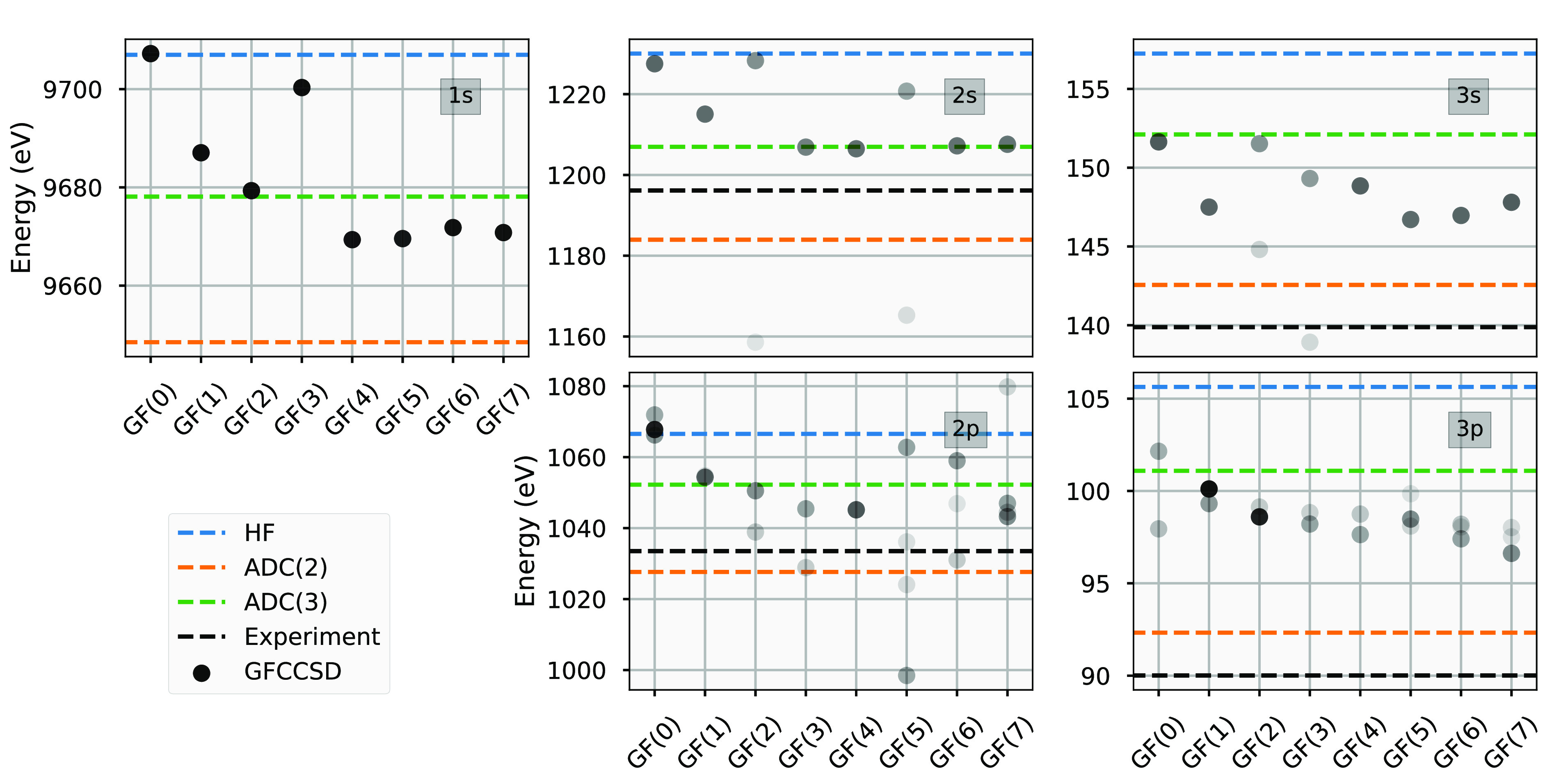
Convergence of CCSD core-level ionization energies from *K*- (1*s*), *L*- (2*s*, 2*p*) and *M*- (3*s*, 3*p*) shells of a zinc atom with increasing moment constraints, in a cc-pwCVTZ basis with X2C Hamiltonian. The transparency in the shading of points is proportional to the weight of the resulting ionization potential on the atomic orbital of the same character as the desired excitation. This shows that different moment constraints can sometimes result in a splitting of the state across a range of energies. The ADC(2) and ADC(3) results are taken from ref (77) and experimental results from ref (76). We note that experimental values for the 2*p* and 3*p* orbitals are obtained by averaging ionization energies for states with the total angular momentum quantum number *J* = 1/2 and *J* = 3/2.

One area where high-accuracy prediction of energy levels is increasingly important is core-level spectroscopy. Similar to what is in Figure 2, we will see that the convergence of core levels with respect to moment expansion is slower and less reliable than the frontier states about the chemical potential, which also can suffer from loss of numerical accuracy and maintenance of biorthogonality at higher moments. In Figure 3 we consider the core energy levels from the *K*-, *L*- and *M*-shells of a zinc atom in a large cc-pwCVTZ basis,^74^ with a two-component X2C relativistic Hamiltonian.^75^ We compare to the experimental results of ref (76) and recent ADC(2) and ADC(3) calculations,^77^ without making the common core–valence separation approximation.^78,79^ We also show the energy of all relevant ionization potentials, where the shading indicates the weight of the excitation on the atomic orbital with the same character. It can be seen that, while sometimes the excitation level is clearly defined and quasiparticle-like, with the state being dominated by a single atomic orbital, for other cases we find that the state can be split into multiple states over a range of relevant energies, with lower weights on the atomic orbital of relevance.

This splitting of excitations could be physical, driven by the correlations inducing an effective broadening of the quasiparticle lifetimes and other satellite features, or it could be an artifact from not fully converging with respect to moment order, or it could be a numerical feature of finite precision arithmetic. It should be stressed that the moments of the final Green’s functions match the original moments provided, with these deep energy-scales not directly probed by the method. Rather, these energy levels have to be resolved via constraining the moments of the overall spectrum at all energy scales, and there are often multiple ways which this can be satisfied. However, at the higher moment orders it does seem as though reasonable convergence and accuracy of these core states are achievable, with the scatter of the larger-weighted states generally below the difference in energy in going from an ADC(2) to ADC(3) level of theory. Despite this, the precise energy of these core states is not fully converged with respect to moment order in all cases, which is exacerbated by the numerical uncertainties which can result from these high moment orders and wide energy scales arising from low-energy states and large basis sets. Further improvements will aim to improve this stability to go to higher moment orders in the future.

### 4.3 A “Ground State” Approximation to the Spectrum

In Figure 2, we also show an alternate approximation denoted GF(0′). In this, the first two particle and hole CCSD GF moments (*m* = 0 and 1) are computed not from an EOM approach of eqs 24 and 25, but instead are found directly from the one- and two-body reduced density matrices (RDMs) of the ground state, which are readily available from many codes (and indeed from different levels of theory). Appendix A details how the *m* = 1 moment can be derived from these RDMs, and how the “commutator trick” effects the rank reduction required for their computation. This derivation is rigorous for variational methods, and will only approximately hold for coupled-cluster theory. Nevertheless, this shows how a limited level of information about the spectrum of charged particle fluctuations can be obtained from these RDMs. This approximation is however fundamentally different at the CCSD level of theory compared to the GF(0) approximation, since the GF moments of eqs 24 and 25 are computed via RDMs in the *absence* of the projection operators (*P*_*X*_ and *P*_*Y*_) onto the 2*p*1*h* and 2*h*1*p* spaces in which the CC Hamiltonian is represented. In this way, computing the moments from the RDMs allows for limited fluctuations into the 3*p*2*h* and 3*h*2*p* spaces which are absent in a CCSD treatment.

The consequence of this is that the spectrum from the GF(0′) approximation has some favorable properties. Chiefly, these are that the energy computed from the spectrum will be equal to the CCSD energy via the Migdal-Galitskii formula^58^—a limitation of traditional CCSD Green’s functions which has been noted from early in their formulation.^28,29^ However, despite allowing for a greater flexibility in the description of the fluctuation space of the excitations, this does not necessarily translate into an improved description of the spectrum or gap compared to GF(0), as seen in Figure 2 and Table 1. This is because there is an imbalance in the implicit description of the ground state compared to the excitations, and it lacks a consistent truncation for effective error cancellation in energy differences (noting however that comparison to traditional CCSD is not an appropriate comparison given the difference in their ansatz for the excitations, but we do not think that this accounts for the majority of the discrepancy). For this reason, all moments are computed with the projections of  into the relevant space to be consistent with the EOM-CCSD description, and include the GF(0′) results only as an interesting comparison and approach to spectral properties from ground state information.

**Table 1:** Mean Absolute Errors (MAE) and Standard Deviations (STD) of the GW100 Test Set for a Series of Methods, Compared to References Values at the ΔCCSD(T) Level of Theory^a^

	IP		EA	
Method	MAE	STD	MAE	STD
HF	0.627	0.708	0.982	0.470
PBE	3.996	1.226	3.071	0.899
GF(0′)^b^	0.226	0.197	1.461	0.904
GF(0)	0.187	0.165	1.528	0.889
GF(1)	0.100	0.122	0.730	0.467
GF(2)	0.084	0.114	0.507	0.326
GF(3)	0.079	0.113	0.365	0.245
GF(4)	0.078	0.114	0.254	0.174
GF(5)	0.072	0.111	0.202	0.169
EOM-CCSD	0.066	0.108	0.050	0.084
ADC(2)	0.661	0.949	0.331	0.474
AGF2^c^	0.437	0.407	0.222	0.160
*G*_0_*W*_0_@HF^d^	0.307	0.263	0.188	0.206
*G*_0_*W*_0_@PBE^d^	0.665	0.328	0.243	0.248

a Shown are the errors at mean-field Hartree–Fock (HF) and PBE density functional theory approximations, as well as the different CCSD GF moment truncations, along with the target EOM-CCSD errors and a number of additional methods employed in quantum chemistry to compute charged excitations.

b The GF(0′) data is missing the largest 10 systems due to memory constraints in storing the 2RDM (which could be alleviated with a more efficient implementation).

c The AGF2 data is missing hexafluorobenzene and xenon due to convergence issues.^15^

d The *G*_0_*W*_0_ data is missing the 8 systems which require an effective core potential (ECP) which was not available in the Fiesta code used to compute them at the time of data collection.^84−86^ The ΔCCSD(T) reference IP and EA values were calculated using the ORCA code.^87,88^ All other values were computed using the PySCF programming package.^67,68^

This approach is formally equivalent to the EKT-1 approach,^80−83^ where the extended Koopman’s theorem (EKT) is employed to produce ionization potentials and electron affinities using the first two (zeroth and first) moments of the CC Green’s function for the hole and particle sectors, respectively. Both approaches construct a spectrum consisting of the same number of poles (*N* in each of the hole and particle sector, where *N* is the number of orbitals), and both methods similarly conserve the first two moments, giving the same spectrum. However, we note that the higher order EKT-*n* are *not* equivalent to the higher moment constraints of GF(*n*) which we detail in this work.

### 4.4 GW100 Test Set

**Figure 4:**
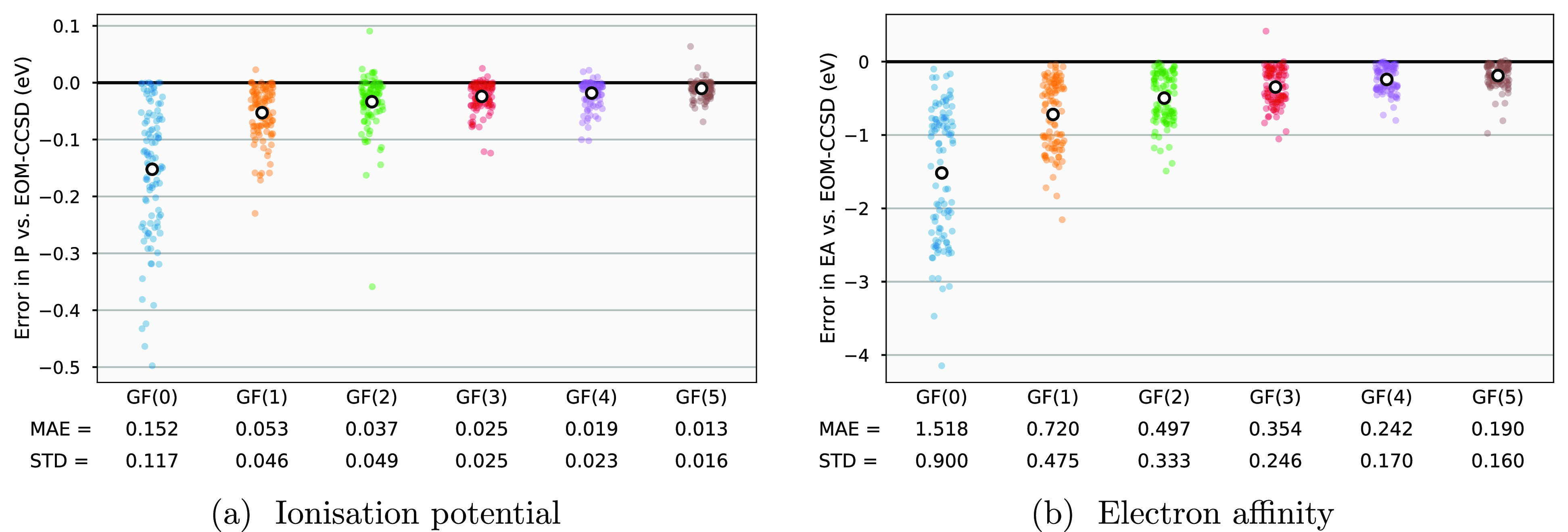
Convergence of the moment-conservation approach to approximate the IP/EA-EOM-CCSD excitations for (a) ionization potential and (b) electron affinity over the GW100 benchmark test set with a def2-TZVPP basis set. The white circle defines the mean absolute error at each order, with the standard deviations in these quantities also given below the plot (in units of eV). Excitations with a weight of <0.1 in the physical space were rejected in order to compare only quasiparticle-like excitations. The EOM-CCSD reference values were calculated using the PySCF package.^67,68^

For broader and more reliable conclusions as to the convergence and accuracy of the GF moment expansion, Figure 4 shows the error in the ionization potential (IP) and electron affinity (EA) of molecules in the GW100 test set compared to the state-specific convergence of the respective “infinite-moment” limit of IP/EA-EOM-CCSD values.^54^ The systems were treated with a def2-TZVPP basis set with the corresponding effective core potential (ECP) applied to Rb, Ag, I, Cs, Au, and Xe to treat core electrons. The errors shown do not reflect the intrinsic error of the EOM-CCSD approximation for these excitations, and quantify only the convergence of the approximation to the lowest-energy excitations from the block Lanczos recursion of section 3 to the true EOM-CCSD poles. Since the accuracy of this approximation relative to the inherent error of EOM-CCSD is important in judging the rate of convergence, in Table 1 we show the aggregated errors between the methods and an accurate ΔCCSD(T) benchmark to include the intrinsic error compared to other perturbative and DFT quantum chemical methods for excitations.

We find errors in the first EA over this test set of Figure 4 to be significantly larger than those of the IP, by an order of a magnitude. This is rationalized by the fact that with any sufficiently large basis set, there will typically be many more virtual orbitals than occupied, and therefore there are considerably more poles in the virtual part of the spectrum, which will also cover a substantially larger energy window. As a result, the convergence of any *single* excitation (e.g., the EA) is harder to achieve with the GF moments defined as integrated over all excitations, and with a larger number of true poles compressed into the same number as defining the occupied pole resolution for the same moment order. We note that there is no necessity in the algorithm to define the same moment truncation for the occupied and virtual spaces, and it is simple to specify a higher order in the IP or EA spaces as desired (including entirely excluding the IP or EA excitations if not of interest).

The intrinsic error (compared to ΔCCSD(T)) of the EOM-CCSD method over the GW100 test set is 0.066 eV for the IP and 0.050 eV for the EA, which characterizes the aim for convergence of the moment expansion in these quantities. Even with a single iteration of the recursion, defining GF(1), the mean absolute error introduced by the moment approximation to the excitations is already less than this intrinsic error for the IP, however the larger errors for the EA mean that the intrinsic error is not met up to GF(5). Despite this, for both the IP and EA there is a clear and systematic convergence in the error with increasing numbers of moments. In comparison to other perturbative approximations to excitations in quantum chemistry, even at GF(0) the error in IP compared to CCSD(T) outperforms ADC(2), AGF2, and *G*_0_*W*_0_ due to its accurate approximation to the superior underlying EOM-CCSD description. For the GF(5) approximation, the error in the EA is similar to the AGF2 method, and only surpassed by the *G*_0_*W*_0_@HF (and EOM-CCSD) approximations, noting that this improvement of *G*_0_*W*_0_ does not extend to a PBE reference due to the strong reference dependence of the *G*_0_*W*_0_ approximation.^50^

In defining these specific frontier excitation energies rather than the overall spectrum, we note that the presented algorithm can suffer from the appearance of low-weighted excitations in the spectrum of eq 41 around the Fermi energy, but which do not “adiabatically connect” to physical excitations in the exact limit of EOM-CCSD. While these spectra will still conserve the input moments, the lack of imposing any explicit excitation structure does not preclude these erroneous excitations, which correspond to poles which are almost entirely located in the “external” space, and are only weakly coupled to the “physical” space. Given their low weight and character far away from traditional frontier quasiparticles, these excitations can generally be identified and removed with an appropriate threshold when aiming to characterize individual frontier excitations. We therefore apply a threshold of a spectral weight of 0.1 in order to define the IP and EA here, which helps remove a small number of these erroneous excitations.

Another point to note is the potential for complex eigenvalues of  with small imaginary components, manifesting in noncausality in the spectrum of eq 41, which is not precluded in CC theory.^89^ The potential for these appears to increase as one increases the number of conserved (non-Hermitian) CCSD moments. In the case of the GF(2) IPs of Figure 4, there exists a significant outlier (the water molecule), whose spectrum correctly finds an excitation energy at an appropriate energy, but where it does not correctly assign a good weight to this excitation and which is therefore removed by this threshold. These numerical aspects emphasize that care needs to be taken when using the approach for the convergence of state-specific excitations in some cases, with the particular strength of the method being in efficiently and systematically resolving the overall spectral intensities over large energy ranges.

## 5 Conclusions

We have presented an efficient and systematically improvable approximation for obtaining the coupled cluster Green’s function which is rapidly convergent to the full resolution over all frequencies in a non-state-specific fashion, and requiring significantly fewer floating-point operations than more traditional approaches. The solver is based on a modified block Lanczos recursion to directly conserve the particle and hole moments of a resulting Green’s function, further adapted for use with the non-Hermitian spectral moments of CC theory. We have shown an application of the approach for construction of full frequency Green’s functions with spectral moments applied from the level of CC singles and doubles; however, we stress that the recursion can equally well be applied to other levels of theory. The convergence has been demonstrated using a series of full-frequency spectra compared to a traditional implementation, and by demonstrating the convergence in the first ionization potential and electron affinities of a large and diverse benchmark set.

The generality of the algorithm to build Green’s functions via moment constraints will mean that future work will look to apply this approach using other methods to compute the GF moments, including different levels of coupled cluster,^27^ stochastic methods,^90,91^ and *GW*.^50^ Additionally, the numerical stability and maintenance of biorthogonality within the recursion will be looked to be improved, allowing effective convergence to the full-frequency limit and individual excitations. The present algorithm should also be readily applicable to *ab initio* solids using a *k*-space resolution, where it can be combined with interpolation schemes to efficiently produce well-resolved spectra in solid state systems. Finally, adaptations toward optical excitations within a similar “static” moment-based framework will also be explored as an alternative perspective for efficient spectral methods.^92^

## Appendix

### Derivation of First-Order Moments from RDMs

For an arbitrary Hamiltonian with one- and two-body operators

49

One can derive an expression for the first-order hole and particle moments in terms of the one- and two-body reduced density matrices. The first moment of the hole Green’s function can be written (for a variational and Hermitian theory) as

51

Using the identity

53

we can expand the one-body contribution to the commutator as

54

and thus

55

Using the identity

58

we can expand the two-body contribution to the commutator as

59

and thus

60

Combining the one- and two-body terms for the shifted Hamiltonian , we arrive at an expression for the first-order hole moment

63

A similar derivation can be found for the *m* = 1 moment of the particle Green’s function as

64

Using the identity

66

we can expand the one-body contribution to the commutator as

67

and thus

68

Using the identity

72

we can expand the two-body contribution to the commutator as

73

Using the identity

75

we can further expand this as

79

The first two terms are identical within a sign via permutational symmetry of the two-electron integrals under exchange of the dummy labels *p* and *q*, leading to

81

Combining the one- and two-body terms for a normal-ordered Hamiltonian we arrive at an expression for the first-order particle moment

82

These expressions are in agreement with those of ref (83).
